# Using X-Ray In-Line Phase-Contrast Imaging for the Investigation of Nude Mouse Hepatic Tumors

**DOI:** 10.1371/journal.pone.0039936

**Published:** 2012-06-28

**Authors:** Qiang Tao, Dongyue Li, Lu Zhang, Shuqian Luo

**Affiliations:** 1 School of Biomedical Engineering, Capital Medical University, You An Men, Beijing, China; 2 School of Medical Image, Tianjin Medical University, Tianjin, China; National Cancer Institute, United States of America

## Abstract

The purpose of this paper is to report the noninvasive imaging of hepatic tumors without contrast agents. Both normal tissues and tumor tissues can be detected, and tumor tissues in different stages can be classified quantitatively. We implanted BEL-7402 human hepatocellular carcinoma cells into the livers of nude mice and then imaged the livers using X-ray in-line phase-contrast imaging (ILPCI). The projection images' texture feature based on gray level co-occurrence matrix (GLCM) and dual-tree complex wavelet transforms (DTCWT) were extracted to discriminate normal tissues and tumor tissues. Different stages of hepatic tumors were classified using support vector machines (SVM). Images of livers from nude mice sacrificed 6 days after inoculation with cancer cells show diffuse distribution of the tumor tissue, but images of livers from nude mice sacrificed 9, 12, or 15 days after inoculation with cancer cells show necrotic lumps in the tumor tissue. The results of the principal component analysis (PCA) of the texture features based on GLCM of normal regions were positive, but those of tumor regions were negative. The results of PCA of the texture features based on DTCWT of normal regions were greater than those of tumor regions. The values of the texture features in low-frequency coefficient images increased monotonically with the growth of the tumors. Different stages of liver tumors can be classified using SVM, and the accuracy is 83.33%. Noninvasive and micron-scale imaging can be achieved by X-ray ILPCI. We can observe hepatic tumors and small vessels from the phase-contrast images. This new imaging approach for hepatic cancer is effective and has potential use in the early detection and classification of hepatic tumors.

## Introduction

Malignant hepatic tumors are tumors that result in high rates of morbidity and mortality. Improvement of therapeutic efficacy and the survival rate from hepatic tumors is an important problem, and the key issue is early diagnosis of hepatic tumors. Conventional methods of imaging diagnosis for hepatic tumors, such as sonography [1]–[6], magnetic resonance imaging (MRI) [5]–[10], computed tomography (CT) [5], [6], [9], [11], digital subtraction angiography (DSA) [11], X-ray microtomography, X-ray fluorescence imaging [12] and other methods, have relatively high rates of diagnostic accuracy for advanced tumors, and their imaging resolutions are only at the millimeter level. However, the early diagnosis of hepatic tumors, especially in sub-clinical diagnosis, may be more difficult. It is easy to overlook small liver tumors of diameters less than 1 mm. Needle biopsy is the most commonly used method for some disease diagnoses. However, this technique is invasive [13], and some small hepatic tumors may be overlooked by needle biopsy, which results in serious consequences because the best time for treatment is missed.

There are many other areas of research in the early detection of hepatic tumors. For example, gold nanoparticles in material science are studied as a hepatic tumor contrast agent [14] to enhance images for the location of nanometer-scale hepatocellular carcinomas. Low-angle X-ray scattering is applied in the detection of structural changes in the serum proteins of patients [15]. This method may detect and treat early hepatic tumors. X-ray ILPCI is a type of imaging that is being researched for detecting early hepatic tumors.

As a new imaging method, X-ray phase-contrast imaging (XPCI) has high spatial resolution and density resolution, which can provide high contrast images by using the phase shift of the X-ray. The density resolution for C, H, O and other light elements is approximately 1,000 times higher than that of traditional X-ray absorption imaging. This technique can greatly improve the image quality of soft tissues, particularly at the interface of tissues, where the refractive index changes significantly [16]–[17]. Therefore, soft tissue imaging using XPCI has some potential in clinical applications. The phase-contrast depends on X-ray coherent scattering rather than absorption; consequently, XPCI can reduce potential radiation damage to tissues [18]. Recently, this technique has been widely used by researchers for imaging small animals. XPCI has four approaches: X-ray interferometer [19], diffraction enhanced imaging (DEI), ILPCI and X-ray grating interferometer [20]. X-ray ILPCI [21] has become a major focus of current research. Its imaging conditions are the simplest, making it more suitable for clinical applications than other methods [22]. X-ray ILPCI has previously been used to study the soft tissues of both humans and small animals, such as mice, rats, rabbits and hamsters [19]. The results have been satisfactory, and high resolution images have been obtained [23]. X-ray ILPCI may be an alternative method for observing hepatic tumors without contrast agents, and it is totally noninvasive. The micro-CT image resolution of mouse liver tumors in animal experiments is 9 μm when a contrast agent is injected [24]. The X-ray ILPCI image resolution of nude mouse liver tumors can be 3.7 μm without a contrast agent. MRI is limited by the magnetic strength, so its spatial resolution is difficult to increase. X-ray fluorescence imaging mainly images the fluorescence of heavy metals in biological tissues, so the heavy metal content of the tissue determines the effect of the X-ray fluorescence imaging. X-ray fluorescence imaging [25] mainly reflects the heavy metal content rather than the specific shape of the tumor, and it does not clearly detect blood vessels. X-ray ILPCI can allow the observation of the tumor shape and the blood vessel distribution around a tumor. X-ray ILPCI and X-ray fluorescence imaging are still in the experimental stages, and it may be a long time before research advances to the clinical application stage.

Texture is the visual perception of local features on an image. Texture analysis of medical images is a sophisticated computer-aided technique that allows the detection of mathematical modes in the gray level distribution of pixels in digital images. It provides an objective characterization of the signal behavior of anatomical structures or pathological processes. The texture features of an image reflect the spatial distribution of the pixel properties, and they usually have irregular local and regular macroscopic characteristics. The texture features of a part of an image are closely related to the gray value changes in this region. A smooth region of an image contains pixels whose gray values are similar to one another, whereas the gray values of the pixels in a rough region differ dramatically [26]. Images need to be preprocessed before the extraction of texture features. Image preprocessing does not require prior knowledge and does not affect the subsequent extraction of texture features. Texture parameters are extracted as image features and calculated quantitatively. Combined with some classification algorithms, such as SVM, back propagation (BP) neural network, etc., texture parameters can distinguish different biological tissues or different pathological conditions within the same biological tissues [27]. When texture features based on GLCM and DTCWT are quantitatively analyzed, normal regions and tumor regions in an X-ray ILPCI image can be distinguished, and different stages of hepatic tumors can be classified by the SVM technique.

## Materials and Methods

### Preparation of animal samples

Nude mice are usually considered to be the laboratory animal offering the closest approximation to human chromosomes. Animal grouping and transplanting: male nude mice, weighing approximately 18 g, were obtained from the Institute of Laboratory Animal Sciences, Cancer Institute and Hospital, Chinese Academy of Medical Sciences. A total of 24 nude mice were grouped randomly into 4 groups. Each group (consisting of 6 mice) was sacrificed after either 6, 9, 12, or 15 days after inoculation with hepatocellular carcinoma cells.

Each nude mouse was anesthetized with an injection of 0.81 mg (45 mg/kg) pentobarbital sodium. After it was anesthetized, we opened the abdominal cavity of the mouse and implanted BEL-7402 human hepatocellular carcinoma cells [28] into its liver. The operation lasted approximately ten minutes, and then the wound was sutured. After approximately two hours, the mouse awoke. These nude mice were then housed in a standard animal laboratory. There were two days to the animal immune response. After either 6 days, 9 days, 12 days or 15 days, the nude mice were sacrificed. Their livers were cleaned, and the hepatic specimens were fixed in a 10% formalin solution. The study was approved by the Experimental Animal Ethics Committee of Chinese Academy of Medical Sciences.

### Principles of X-ray ILPCI

X-ray ILPCI is called propagation-based imaging or in-line holography, and it is based on the principle of Fresnel diffraction. The Fresnel diffraction formula can be expressed as follows:
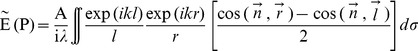
(1)Where, P is any point on the imaging surface, A is the spherical wave amplitude, λ is the wavelength, k is the wave number, l is the distance from the ideal point source to a point on the surface of the object and r is the distance from a point on the surface of the object to P. 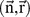 and 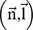 represent the angle between 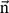 and 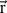 and 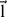, respectively.

The formula is usually shown approximately in actual diffraction problems because of the complex integrand. In addition, the work of many researchers is based on the Wigner distribution theory. First, in the coaxial or paraxial approximation, the inclination factor for the Fresnel integral equation can be set equal to 1 without considering the impact of integration. Second, the distance from a point to the reference point on the viewing screen has a minimal effect on coherence, so the impact of the spherical sub-wave on the reference point can be ignored. After the initial approximation above, the Fresnel diffraction [29] formula can be expressed as follows:
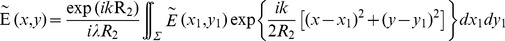
(2)Where, R_2_ indicates the distance from the source to the detector. The incident wave arriving at the front surface of the object is assumed to be a plane wave. If the amplitude is not taken into account, 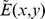 can be simplified into the transfer function q(x, y) of the object, and then the Fourier transformation is applied to both sides of the spatial domain expression (2). The frequency domain expression is as follows:
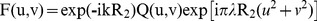
(3)Where, u and v are spatial frequencies. If an object is assumed to be weakly absorptive, the formula (3) can be transformed into the following space domain and frequency domain expressions (4), (5):



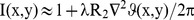
(4)

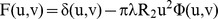
(5)


From the spatial intensity expression, we can see that image visibility is proportional to the Laplacian operator of the X-ray phase shift after passing through an object. Phase variation will be produced when the internal density of an object changes dramatically, and a second-order partial differential equation applied to the phase shift will greatly impact the image contrast. After the X-rays have traveled a certain distance, the intensity distribution received by the detector will reflect the edge enhancement of an object.

**Figure 1:**
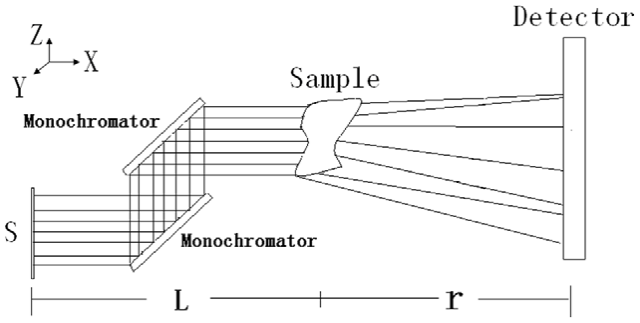
The schematic illustration of the synchrotron radiation in X-ray ILPCI. A synchrotron radiation beam is refracted by two monochromators into a beam of coherent monochromatic light. Fresnel diffraction occurs on the surfaces of different tissues when the monochromatic beam passes through the object. When the distance between the detector and the sample is appropriate, the phase-contrast image is formed.

The schematic diagram of the synchrotron radiation in X-ray ILPCI is shown in Fig. 1.

### Imaging experiments

**Figure 2:**
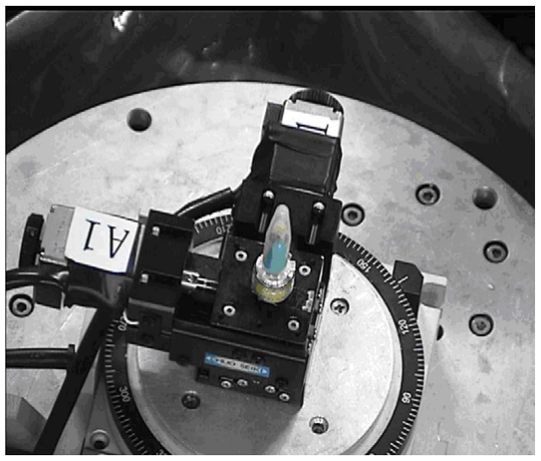
The X-ray ILPCI partial facility of SSRF. This is sample table. The sample can be rotated 180 degrees and is placed in the center. The sample has been wrapped in insulating material.

**Figure 3:**
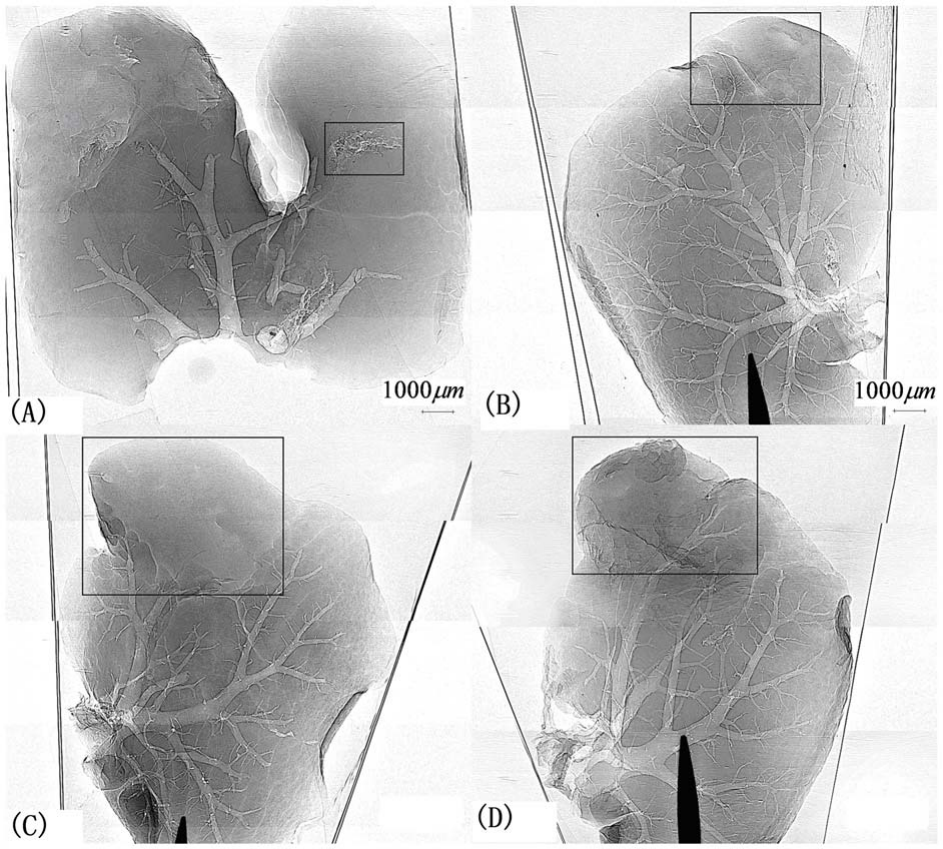
X-ray ILPCI projection images of hepatic tumors at different stages. (A) Image of a 6-day-old hepatic tumor. (B) Image of a 9-day-old hepatic tumor. (C) Image of a 12-day-old hepatic tumor. (D) Image of a 15-day-old hepatic tumor. Images of livers from nude mice sacrificed 6, 9, 12, or 15 days after inoculation with cancer cells are obtained. Images of 9-day-old, 12-day-old, 15-day-old samples show necrotic lumps in the tumor tissue and vessels surrounding the tumors.

The X-ray ILPCI experiments were carried out at the BL13W1 beamline of Shanghai Synchrotron Radiation Facility (SSRF). The X-ray ILPCI facility is shown in Fig. 2. Our project number is 08sr0074. The photon energy ranged between 8 and 72.5 keV. In this study, the monochromators were made of Si (111) crystal. The energy of the coherent monochromatic light formed by the refraction of the synchrotron radiation beam passing through the two monochromators was 15 keV. The sample exposure time was 6 ms. The resolution of CCD detector was 9 μm. The distance between the samples and the CCD was 50 cm. Projection images of the hepatic tumors from nude mice are shown in Fig. 3.

To simulate an actual human environment, the hepatic samples were placed in a 10% formalin solution; the projection imaging results are not ideal. The blood vessels of the liver are not clear. However, when we take the sample out of the formalin solution and image directly without any other substances, we find that the results clearly show the texture and the presence of tumors and blood vessels.

Specifically, nude mouse hepatic specimens containing the transplanted BEL-7402 human hepatocellular carcinoma cells were wrapped with insulating material and were placed on the sample table. The specimens were vertically and horizontally adjusted to allow matrix scanning and the capture of projection images.

### PCA of the image texture based on GLCM

To further investigate the texture features, the textures of X-ray ILPCI projection images were quantified using the GLCM method [26]. GLCM is the measurement of an image's statistical properties, visual characteristics, information theory measures and correlation based information. In this paper, we use nine texture parameters based on GLCM. They are the angular second moment, inertia, inverse difference moment, entropy, correlation, sum average, difference average, sum entropy and difference entropy. The gray level co-occurrence matrix is defined as Cij.

The formula for the angular second moment can be expressed as follows:
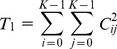
(6)


The formula for the inertia can be expressed as follows:
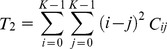
(7)


The formula for the inverse difference moment can be expressed as follows:
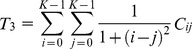
(8)


The formula for the entropy can be expressed as follows:
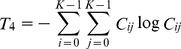
(9)


The formula for the correlation can be expressed as follows:
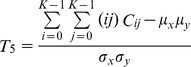
(10)


The formula for the sum average can be expressed as follows:
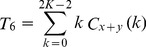
(11)


The formula for the difference average can be expressed as follows:
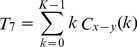
(12)


The formula for the sum entropy can be expressed as follows:
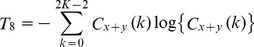
(13)


The formula for the difference entropy can be expressed as follows:
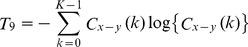
(14)


The nine texture parameters reflect the uniformity, randomness or other features of an image. A total of 20 square regions of interest (ROIs) were selected in each tumor region of different stage hepatic refraction image. In addition, the 20 ROIs were placed within each normal region of the same stage hepatic refraction image. Each ROI had a size of 60×60 pixels. Subsequently, the texture features of the normal tissue and tumor tissue regions were extracted separately.

The principles of PCA can be expressed as follows: first, we perform standardization processing on the matrix consisting of the nine texture parameters of the 20 ROIs. Then, the correlation coefficient matrix of the texture matrix, the eigenvalues denoted as [λ1, λ2, λ3…λ9] of the correlation coefficient matrix, and the eigenvector denoted as [ei1, ei2, ei3…ei9] of λi, are solved. The new component can be expressed as follows:
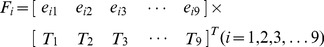
(15)


The contribution rate (CR) of Fi can be expressed as follows:
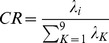
(16)


If the CR of Fi is greater than some threshold, we use Fi as the principal component.

PCA was carried out on the texture parameters we acquired above. The components with the CR up to 80% were used as the principal components. Subsequently, the means and standard deviations of the principal components were computed to discriminate the normal and tumor regions.

### PCA of the image texture based on DTCWT

We used DTCWT to decompose the images into two levels and chose ROIs. DTCWT was carried out on each ROI. After the deccomposition, we acquired two low-frequency coefficient images and two high-frequency coefficient images. The texture features of the four images in Fig. 3 were also extracted. Finally, we analyzed the principal components (PC) and computed their means and standard deviations.

### Tumor classification using SVM

In this paper, SVM is used to classify hepatic tumors into different stages. The low-frequency coefficients of wavelet decomposition carry abundant information about the tumor tissue, so the first-level texture parameters of the low-frequency decomposition are used as the basis for classification. The acuqired texture features of the ROIs for each tumor stage were analyzed by PCA, and the components with the CR greater than 80% were used as the PCs.

## Results

### X-ray absorption imaging of hepatic tissue

**Figure 4:**
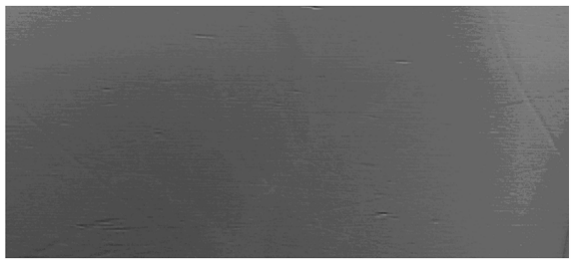
X-ray absorption image of nude mouse normal liver tissue. The image resolution is low and the tissue texture characteristics are not distinct.

The traditional X-ray absorption imaging of normal hepatic tissue is shown in Fig. 4. In Fig. 4, we find that the blood vessels are not very obvious and that the tissue texture characteristics are not distinct. The traditional X-ray absorption imaging is dependent on the X-ray absorption attenuation coefficient. After X-rays penetrate the object, they are absorbed, and their energy declines. The attenuation coefficient of the X-rays is changed. Thus, the traditional X-ray absorption imaging of soft tissue is relatively poor.

### Imaging results of X-ray ILPCI

Experimental samples of hepatic tumors were imaged using a CCD detector with an image resolution of 9 μm. The livers were removed from 10% formalin.

Fig. 3 shows projection images from X-ray ILPCI, and we can clearly observe the changes in the different stages of hepatic tumors and their subtle structures. The image clarity of X-ray ILPCI is better than that of the X-ray absorption imaging shown in Fig. 4. The regions of the images delineated by rectangles are tumor tissues, which have been identified by a clinical expert. Images of livers from nude mice sacrificed 6 days after inoculation with cancer cells show diffuse distribution of the tumor tissue, but images of livers from nude mice sacrificed 9, 12, or 15 days after inoculation with cancer cells show necrotic lumps in the tumor tissue. Vessels can be seen surrounding the tumors from nude mice sacrificed 9 or 15 days after inoculation with cancer cells. From Fig. 3 (C), we can see that there are vessels under the tumor in order to supply it with enough blood.

## Discussion

### Results of PCA of the texture features based on GLCM

The PCA of the texture features based on GLCM was carried out on the four images in Fig. 3. All of the CRs of the first PC F1 of the samples are above 80%, and the other components' CRs are less than 10%. Thus, we used F1 as the basis for distinction between normal regions and tumor regions. The means, standard deviations and CRs of F1 for different samples are shown in Table 1. The parameters of Normal(F1) have small means with large standard deviations (for example: 0.851±0.128), but these of Tumor(F1) have large means with small standard deviations (for example: −2.031±0.014). It can also be observed that the F1 of normal regions is positive and the F1 of tumor regions is negative. There is a significant difference between the tumor and the normal regions. They can be clearly distinguished through quantitative analysis of texture features based on GLCM.

**Table 1:** Results of the PCA of texture features based on GLCM.

	6d	9d	12d	15d
**Normal (F_1_)**	1.427±0.077	1.430±0.051	1.632±0.041	0.851±0.128
**Tumor (F_1_)**	−1.592±0.063	−1.760±0.011	−2.104±0.018	−2.031±0.014
**CR**	91.57%	83.27%	83.14%	80.38%

ROIs are randomly selected by the operator. Each time, it is different F1 and CR values when operator calculates the different ROIs. However, this selection does not change the positive or negative value of F1 for a normal or tumor region. If F1 is greater than zero, it indicates a normal region. If F1 is less than zero, it indicates a tumor region. Thus, randomly selecting ROIs does not directly impact the detection of tumors. The selection of different ROIs does not directly affect the diagnosis of early hepatic tumors.

### Results of PCA of the texture features based on DTCWT

The PCA of the texture features based on DTCWT was carried out on the projection images of the 6-, 9-, 12- and 15-day-old samples. Because of the high CR, we also simply took the first PC F1 as the basis for the classification of normal regions and tumor regions. The means, standard deviations and CRs of F1 for different samples are shown in Table 2, where, HF1 is the first PC of high-frequency coefficients in level 1, HF2 is the first PC of high-frequency coefficients in level 2, LF1 is the first PC of low-frequency coefficients in level 1 and LF2 is the first PC of low-frequency coefficients in level 2.

**Table 2:** Results of the PCA of texture features based on DTCWT.

		HF1	HF2	LF1	LF2
**6d**	**Normal**	2.092±0.037	2.062±0.041	1.982±0.092	2.003±0.099
	**Tumor**	−0.965±0.114	−0.864±0.012	−1.317±0.081	−1.162±0.083
	**CR**	87.79%	87.75%	93.74%	94.69%
**9d**	**Normal**	1.534±0.096	0.989±0.083	1.034±0.096	1.100±0.099
	**Tumor**	−0.109±0.012	0.142±0.011	−0.635±0.096	−0.569±0.098
	**CR**	87.51%	84.63%	88.37%	94.13%
**12d**	**Normal**	2.738±0.008	2.409±0.028	1.170±0.102	1.303±0.104
	**Tumor**	−1.783±0.071	−1.854±0.091	−0.189±0.003	−0.083±0.001
	**CR**	88.93%	88.46%	87.49%	94.39%
**15d**	**Normal**	2.668±0.007	2.202±0.025	0.889±0.011	0.982±0.011
	**Tumor**	−0.295±0.015	−0.646±0.136	0.126±0.018	0.179±0. 001
	**CR**	88.43%	87.52%	84.39%	90.75%

**Figure 5:**
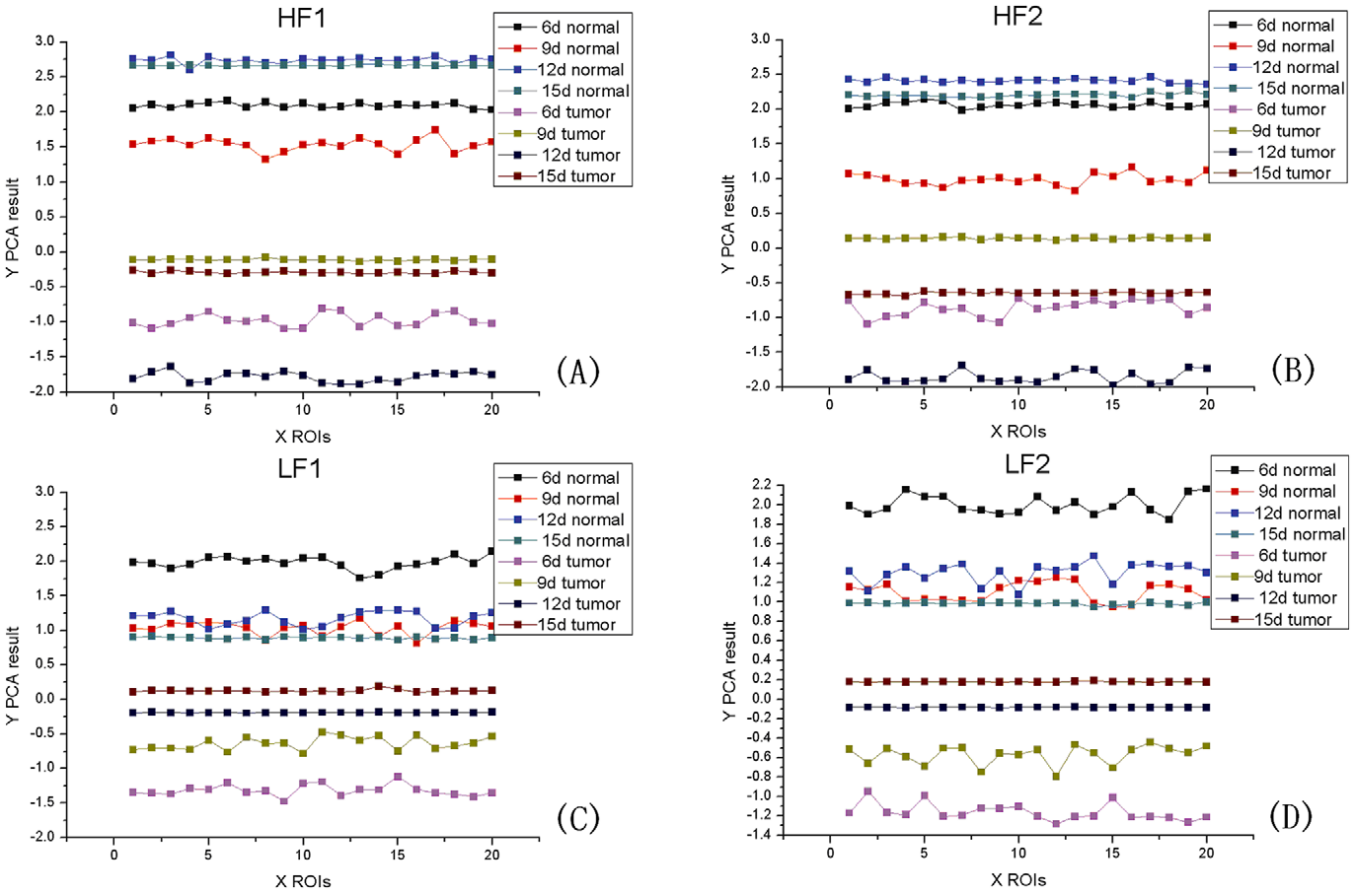
Results of the PCA of texture features based on DTCWT. The PCA of the all the randomly ROIs' texture features based on DTCWT was carried out on the projection images of the 6-, 9-, 12- and 15-day-old samples. Results of the PCA of the texture features are (A) HF1, (B) HF2, (C) LF1 and (D) LF2.

Fig. 5 shows the PCA results of all the randomly ROIs' texture features based on DTCWT. Fig. 5 (A), (B), (C) and (D) show the PCA results of HF1, HF2, LF1 and LF2. From these figures, we can see that the texture features values of the normal regions are greater than those of the tumor regions. Because the low-frequency coefficient images carry abundant information about biological tissues, the values of the texture features of tumor regions increase monotonically with the growth of the tumors, as shown in Fig. 5 (C) and (D). However, high-frequency coefficient images carry less information than low-frequency coefficient images, so we can not see the monotonicity in Fig. 5 (A) and (B). The PCA results of LF1 and LF2 provide a favorable basis on which to judge the different stages of the tumors.

### Results of tumor classification using SVM

In this paper, we randomly selected 20 ROIs from each image of a 6-, 9-, 12- or 15-day-old hepatic tumor. The DTCWT decomposition was carried out on each ROI. We extracted the texture features of the decomposition of coefficient matrix on a low-frequency level 1 and analyzed the PC. The CR of the first PC F1 was 89.02%. The experimental result shows that the maximum classification accuracy for classifying hepatic tumors into different stages was 83.33%.

Cancer diagnosis is the result of a comprehensive diagnosis, and biopsy remains the gold standard for the diagnosis of tumors. The analysis method of texture features reported in the paper is one of the auxiliary means of the tumor diagnosis. Texture features of these suspected cancer cases have the same texture features reported in this article, but other diagnostic tests such as blood tests and biopsies are still needed. Biopsy is a necessary and sufficient test for early diagnosis of hepatic tumors. In this article, hepatic tumors of samples have been known to exist before the X-ray ILPCI experiments were performed.

The lesion can been observed in a X-ray ILPCI projection image, and the texture characteristics have the same features as those reported in this article, but the final diagnosis of hepatic cancer still requires a biopsy. This paper presents a noninvasive auxiliary method for the early diagnosis of hepatic cancer.

### Conclusion

From the above results, we can see that X-ray ILPCI has high contrast resolution and spatial resolution and that the subtle structure of tissues can be observed with X-ray ILPCI. The spatial resolution of images is improved from the millimeter level to the micron or even sub-micron level by X-ray ILPCI, which has a potential application in the early diagnosis of hepatic tumors. The range of substances that X-ray imaging can detect extends from highly absorbing heavy elements to weakly absorbing light elements, which has some potential clinical applications. Compared with conventional imaging, X-ray ILPCI has greater advantages.

In this paper, we study the morphological changes of early hepatic tumors. Phase-contrast images show that the development of tumors may cause a change in the density of tissues or coarseness in tissue texture, and the seriousness degree of tumor can be discriminated by these morphological features. Using quantitative texture analysis, normal regions and diseased regions in the projection image can be distinguished. The low-frequency coefficient images of DTCWT carry abundant information about biological tissues, whereas GLCM only describes the distribution of pixel pairs in a comprehensive image. Thus, DTCWT is better than GLCM in terms of feature extraction. We also use the SVM method for the first time to classify different stages of hepatic tumors. However, because the number of samples is too small and lacks a certain degree of statistical significance, the accuracy of the classification is not high. In the future, we will increase the number of samples to solve this issue.

X-ray ILPCI is still in the experimental stages, and experiment samples are limited to static samples. There is still no good way to process live samples. The X-ray ILPCI device of SSRF has a small field of view. It takes us approximately half an hour to acquire images from 0 degrees to 180 degrees. Samples must not move within that half an hour. Movement of the sample has a direct impact on the image and may even lead to vision disappeared. It is difficult to solve the problem of movement from the breathing of living samples in vivo experiments. These movements lead directly to artifacts in the final projection images.

It is good effect for a single sample. The image effect may be still clear if the sample has been placed in a water or formalin solution to simulate physiological conditions. We obtained a total of more than 1000 2D projection images using X-ray ILPCI of a nude mouse hepatic tumor sample by rotating it by 180 degrees, and we subsequently used image processing technology such as a filtered back-projection reconstruction imaging algorithm or another reconstruction imaging algorithm. Tomography images can be reconstructed. The overlapping details of 2D projection images can be more clearly observed in 3D tomography images. It can be observed every internal detail in the reconstruction images of the diffraction CT of X-ray ILPCI. Thus, the thickness of the sample does not affect the imaging result.

X-ray ILPCI presents a micron-scale imaging method. X-ray ILPCI requires further research before its clinical application. It will be a qualitative leap, once X-ray ILPCI is used clinically. X-ray ILPCI is an innovation from current millimeter-scale to micron-scale image resolution, and it may represent a new noninvasive auxiliary imaging technique for diagnosing hepatic tumors.
